# Characterizing Interactive Communications in Computer-Supported Collaborative Problem-Solving Tasks: A Conditional Transition Profile Approach

**DOI:** 10.3389/fpsyg.2019.01011

**Published:** 2019-05-08

**Authors:** Jiangang Hao, Robert J. Mislevy

**Affiliations:** Educational Testing Service, Princeton, NJ, United States

**Keywords:** collaborative problem solving, communication, transition matrix, stochastic process, assessment

## Abstract

Communication in a collaborative problem-solving activity plays a pivotal role in the success of the collaboration in both academia and the workplace. Computer-supported collaboration makes it possible to collect large-scale communication data to investigate the process at a finer granularity. In this paper, we introduce a conditional transition profile (CTP) to characterize aspects of each team member's communication. Based on the data from a large-scale empirical study, we found that participants in the same team tend to show similar CTP compared to participants from different teams. We also found that team members who showed more “negotiation” after the partner “shared” information tended to show more improvement after the collaboration while those who continued sharing ideas while their partners were negotiating tended to improve less.

## 1. Introduction

Technology advancement allows computer-supported collaboration to be widely adopted in both academia and the workplace. Compared to face-to-face collaboration, online collaboration significantly reduces the effort and cost of organizing joint work, making it ideal for a wide range of collaborative activities (Stahl et al., 2006). The communication data in computer-supported collaboration contain rich information regarding the collaboration process. Understanding the communication process will help to identify pathways to more successful collaboration outcomes. Such knowledge can further inform the development of real-time facilitation or intervention mechanisms to scaffold the collaboration.

The analysis of communication data (or discourse analysis as it is often called in the computer-supported collaborative learning (CSCL) community) usually starts with the coding or labeling of each turn (or several turns that constitute large speech units) of communications based on a framework (rubrics) being developed to address specific research questions. For example, a number of coding frameworks have been developed to analyze different aspects of the communications among team members, such as the coding framework for collaborative problem solving (CPS) skills (Liu et al., 2015), for the interactive patterns in collaboration (Andrews et al., 2017), for cohesion and language (Graesser et al., 2004; Dowell et al., 2016), and for dialog acts (Allen and Core, 1997). Based on human-coded discourse, natural language processing (NLP) techniques can be employed to automate the annotation to an accuracy level that is close to human coding (Rosé et al., 2008; Rus et al., 2015; Flor et al., 2016; Hao et al., 2017a).

The codings of discourses are numerical representations of the communication data and can be used as input variables for developing higher level feature representations of the communication process, or for developing statistical models of the process. Given that the communication data and codings often involve multiple interacting team members, it is of interest to develop feature variables that characterize both team performance and individual performance. Traditional discourse analysis usually uses the frequency of different codings (e.g., Dowell et al., 2016) or sequence of codings (e.g., Hao et al., 2016) as the high-level representations of the communication. However, such representations fail to capture the information of how a specific member responds to different types of utterances from others throughout the communication process. To address this issue, in this paper, we introduce a conditional transition profile (CTP) approach to form representations of each team member's responses to different types of utterances (based on a given coding framework) from other members. In collaborative work, what one member says is important, but how a member responds to the others' utterances may contain more information about the member's skills in collaboration. The CTP approach provides a quantitative measure of how a team member responds to other team members. To illustrate the effectiveness of the method, we apply the CTP to data collected through a large-scale online collaborative task from the ETS collaborative science assessment prototype (ECSAP) project and show an example of how the team members' CTPs were related to their performance improvements after the collaboration.

## 2. Conditional Transition Profile

Suppose we have a coding framework that has *k* different categories, the *t*-th turn of the communication can be characterized by a *k* dimensional state vector **X**_*t*_, with elements either 0 or 1, indicating whether a given category is assigned to this turn of discourse^1^. For coding frameworks that require mutually exclusive codings, the state vector will have only one element as 1 and all others as 0. The states in a communication process can be considered from both the team level and the individual level. At each level, the most straightforward measure is the cumulative counts of the different states. A CPS profile based on the counts of states at the team level has been introduced to characterize the overall collaboration process of the team (Hao et al., 2016). In this CPS profile, we considered the counts of different states (unigram) and consecutive state pairs (bigram), though the approach can be extended to include the counts of n sequential states (n-gram). It has been shown that different CPS profiles are related to different collaboration outcomes of the team (Hao et al., 2016).

In the current paper, we further generalize the CPS profile from characterizing the whole team process to characterizing each team member's communication process. The most straightforward way to generalize the CPS profile is the direct counts of different states from each team member instead of all the team members. However, in a communication, what one member (target team member) says depends heavily on the other members' preceding discourses. As such, counting the states of a target team member by conditioning on other partners' preceding discourse states should encode more information about the individual's communicative moves in context than merely counting all the states together. As such, we introduce a conditional transition profile for each team member as follows.

For a sequence of coded discourses^2^, we can represent the states of communication in Table 1, where the column name indicates the states of the discourse from the targeted team member and the rows indicate the states of the discourse from the most immediate preceding discourse category from other team members. The numbers in the cells are the counts of the occurrences of the states specified by the corresponding row and column names. It is worth noting that we consider only the most immediate turns of discourses and ignore longer range dependency, though the extension to longer range dependency is straightforward. The reason for doing this is that the majority of short online conversations do not display long range dependency (some empirical evidence of this can be found in Hao et al., 2017a). The elements of a CTP are defined as follows,

(1) $$\begin{array}{l} C\left(D_{i}|{\bar{D}}_{j}\right)=N_{ij} \end{array}$$

where *D*_*i*_ denotes the state (coding category) *i* of the discourse from the targeted team member and ${\bar{D}}_{j}$, denotes the state *j* of the immediately preceding discourse from other team members. Here *i* runs for the columns and *j* runs for the rows. *N*_*ij*_ is the count of occurrences of the state in the corresponding cell. Note that this matrix is very similar to the (weighted) adjacency matrix widely used in graph theory, except that the latter is traceless (Biggs, 1993).

**Table 1:** Conditional transition profile of the communication.

	**State 1**	**State 2**	**State 3**	**⋯**
State 1	*N*_11_	*N*_12_	*N*_13_	···
State 2	*N*_21_	*N*_22_	*N*_23_	···
State 3	*N*_31_	*N*_32_	*N*_33_	···
···	···	···	···	···

*The columns correspond to the states of the discourse from the targeted team member and the rows correspond to the states of preceding discourses from the other team members*.

In many practical applications, the relative ratios of the categories are often considered important. A representation of the ratios can be obtained by normalizing each cell of the table by the sum of its row.

(2) $$\begin{array}{l} T\left(D_{i}|{\bar{D}}_{j}\right)=N_{ij}/\left(\underset{i}{\sum }N_{ij}\right) \end{array}$$

We call this the normalized CTP. In practice, as some elements could be zero due to a small sample size, so smoothing techniques, such as Laplace smoothing (Schütze et al., 2008), can be used to estimate the elements of the normalized CTP as follows,

(3) $$\begin{array}{l} T\left(D_{i}|{\bar{D}}_{j}\right)=\frac{N_{ij}+\alpha }{\sum_{i}N_{ij}+\alpha k} \end{array}$$

where α > 0 is a smoothing parameter. We call the $C\left(D_{i}|{\bar{D}}_{j}\right)$ as conditional transition profile and $T\left(D_{i}|{\bar{D}}_{j}\right)$ as normalized conditional transition profile. Generally speaking, the $C\left(D_{i}|{\bar{D}}_{j}\right)$ contains more information than $T\left(D_{i}|{\bar{D}}_{j}\right)$ as the latter can be derived from the former but not the other way around. $T\left(D_{i}|{\bar{D}}_{j}\right)$ characterizes the probability of the transition among states and could be more generalizable than $C\left(D_{i}|{\bar{D}}_{j}\right)$ under some circumstances. A reliable estimate of the elements in $T\left(D_{i}|{\bar{D}}_{j}\right)$ requires that the number of the occurrences in each cell should be large enough, which suggests that one may want to use the $C\left(D_{i}|{\bar{D}}_{j}\right)$ instead of $T\left(D_{i}|{\bar{D}}_{j}\right)$ if the count numbers are low. In the above definition of the CTP, we consider the counts by conditioning only the most immediately preceding turn by others. One can extend this to higher order association for situations where long-range dependency prevails in the communication.

It is worth noting that the normalized CTP resembles the stochastic matrix (also known as Markov matrix) if the underlying communication process is a discrete time Markov process that meets the following condition (Van Kampen, 1992; Grimmett and Stirzaker, 2001).

(4) $$\begin{array}{l} \text{P}\left({\text{X}}_{t}|{\text{X}}_{t-1},\cdots \;,{\text{X}}_{1}\right)=\text{P}\left({\text{X}}_{t}|{\text{X}}_{t-1}\right) \end{array}$$

where *t* denotes the *t*^*th*^ step of the process. A transition matrix (or stochastic matrix) **P** with elements

(5) $$\begin{array}{l} {\text{P}}_{ij}\left(t\right)=\text{P}\left({\text{X}}_{t}={\text{x}}_{j}|{\text{X}}_{t-1}={\text{x}}_{i}\right) \end{array}$$

will characterize the transition structure of the Markov process. If a Markov process is stationary (homogeneous), e.g., the following equation holds for all *t*, *i*, and *j*:

(6) $$\begin{array}{l} \text{P}\left({\text{X}}_{t}={\text{x}}_{j}|{\text{X}}_{t-1}={\text{x}}_{i}\right)=\text{P}\left({\text{X}}_{1}={\text{x}}_{j}|{\text{X}}_{0}={\text{x}}_{i}\right) \end{array}$$

and we can readily predict the probability of different states for the (t+1)^*th*^ turn based on the preceding turn and the initial turn through the following equation,

(7) $$\begin{array}{l} {\text{X}}_{t}={\text{X}}_{t-1}\text{P}={\text{X}}_{0}{\text{P}}^{t} \end{array}$$

One notable difference between the normalized CTP and the stochastic matrix of Markov process is that the former is not defined on a closed set of states as one team member's states are dependent on other team members' states instead of her own. As such, the (normalized) CTP introduced above is more a way to numerically represent an aspect of the coded communication process for each team member rather than claiming the mathematical properties associated with the stochastic matrix of a Markov process, though some methods based on the stochastic matrix may still be borrowed to analyze the normalized CTP.

In the next section, we will show how the CPT approach can be used to characterize empirical communication data.

## 3. Empirical Study

### 3.1. Task and Data

**Figure 1:**
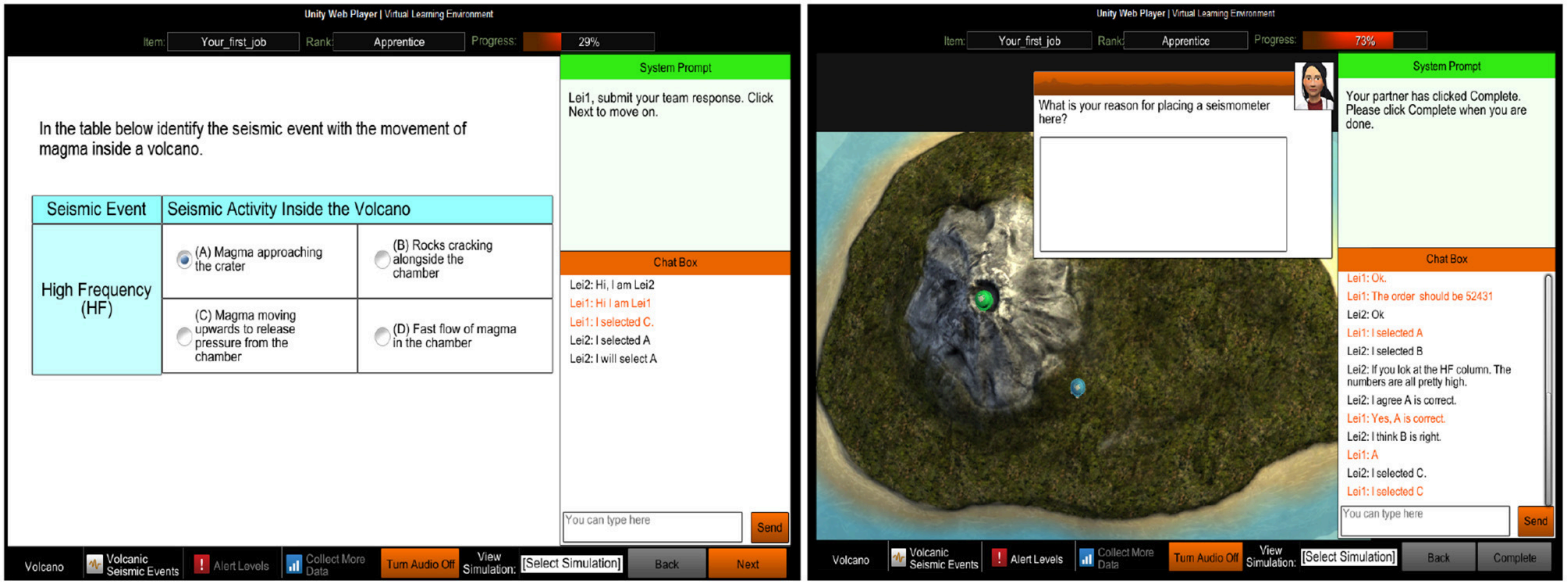
Two screenshots of the simulation-based collaborative task used in the ECSAP.

We carried out the ECSAP project to explore the assessment of communications in large-scale online CPS activities. The goal is to investigate what CPS skills can be detected in the communications and how these skills are related to collaboration outcomes. The details of ECSAP are beyond the scope of this paper, and we refer the readers to Hao et al. (2017b) for a description of the study. The core part of the ECSAP is a simulation-based task that allows two human participants to collaborate through a chat window to complete a set of questions and tasks about volcano science (Hao et al., 2015). Figure 1 shows two screenshots of the simulation-based collaborative task. In the simulation task, the participants were shown some tutorials about the factors related to volcano eruption. Then, they were asked to answer about fifteen questions, during which they need to carry out some small experiments, such as deploying seismometers around a virtual volcano to collect data, to assist them in answering the questions. The first seven questions are selected responses which allow us to impose a set of structured system prompts to maximize the information elicitation. For each of the seven questions, the system prompts each team member to respond individually at first and then prompts the team members to collaborate with each other to discuss their answers via a chat window. After the collaboration, each member is given a chance to revise her initial answer. By checking the difference in the scores on the initial and revised answers, we can calculate each person's gain/loss from the collaboration. The remaining eight questions require manipulation of the tools in the simulation, which makes it more difficult to impose the initial-discuss-revise procedure. They are not addressed in the current analysis. In addition to this simulation-based collaborative task, we also administered a general science knowledge test (Rundgren et al., 2012) to each participant to measure her content-relevant knowledge.

We collected data through a crowdsourcing data collection platform, Amazon Mechanical Turk (Kittur et al., 2008). We recruited 1,000 participants located in the United States with at least one year of college education and randomly assigned them into 500 dyads to complete the simulation-based collaborative task. Seventy-eight percent of the participants were White, 7% were Black or African American, 5% were Asian, 5% were Hispanic or Latino, and 5% were multiracial. Half of the participants are males and half are females, and the age ranges from 25 to 54. Most of the participants have prior experience of online communication, though not necessarily collaborative problem solving. After removing the teams that did not complete the task successfully, we were left with 474 dyads. In each team's response, there are about 80 turns of chat in total and about 30 turns around the first seven questions. We noticed that many teams did not precisely follow the initial-collaborate-revise procedure we set forth and started some non-prompted discussions when they were asked to answer alone. In our analysis, we consider only the teams that have no more than two non-prompted discussions. After this cut, we were left with 237 out of the 474 dyads. The analyses in this paper are based on this subset unless otherwise stated.

The data from each collaborative session include both the responses to the questions in the simulation and the text-chat communication between the team members around each question. The responses to the questions were scored based on the rubrics shown in Zapata-Rivera et al. (2014). We developed a framework for coding the communication data in CPS (Liu et al., 2015) based on CSCL literature and the assessment frameworks from PISA 2015 (Organization for Economic Co-operation and Development, 2013) and ATC21S (Griffin et al., 2012). This framework considers four skills, namely, sharing ideas, negotiating ideas, regulating problem-solving and maintaining communication, which have been identified to be highly relevant to the CPS activity we are targeting. Each turn of the chat communications was coded into one of the four categories of skills based on our CPS framework. Table 2 shows some example chats and states. Two human raters were trained on the CPS framework, and they double-coded a subset of the discourse data (15% of the data). The unit of coding is each turn of a conversation or each conversational utterance. The inter-rater agreement in terms of unweighted kappa is 0.67.

**Table 2:** Example of a part of annotated chat data from one teams.

**Topic**	**Member**	**Chat**	**State**
IntroduceYourselves	A	Hi	Maintaining
IntroduceYourselves	B	Hi, I'm Jennifer	Maintaining
Question1A	A	chose b, cause its rocks cracking that cause the high frequency events	Sharing
Question1A	B	yes, same here	Negotiating
Question1B	A	d sound right to you?	Regulating
Question1B	B	I couldn't remember, I thought it was C	Regulating
Question1B	A	you are right	Negotiating
QuestionsP2	B	A and B?	Regulating
QuestionsP2	A	yes, that's what i got	Negotiating
QuestionsP3	A	52431?	Regulating
QuestionsP3	B	I was only sure about 5 and 1 being first and last	Sharing
QuestionsP3	B	4 is probably second to last	Sharing
ExampleSeisQuestion1	B	A?	Regulating
ExampleSeisQuestion1	A	picked a	Sharing
ExampleSeisQuestion2	A	thoughts?	Regulating
ExampleSeisQuestion2	B	b?	Regulating
ExampleSeisQuestion2	A	same	Negotiating
ExampleSeisQuestion3	A	obviously c	Sharing
ExampleSeisQuestion3	B	c	Sharing
···	···	···	···

*The topic column indicates the specific question around which the conversations happened. The member column indicates which member in the dyadic team produced the discourse*.

### 3.2. Methods

Given that there are about 30 turns of conversations in each team and there are four different coding categories, the expected count in each cell of the four by four matrix is relatively low—about two. Therefore, we choose to use the CTP instead of the normalized version in this paper. The central research question we want to address is the usefulness of the CTP representation of each participant's communication process. As one aspect of this question, we investigated whether such a representation of the communication process is related to the participant's gain or loss as measured based on their total score changes between the initial and revised responses. The hypothesis is that if the CTP is an effective method for characterizing the collaboration process, it should have implications for the collaboration outcomes. We try the following two approaches to gain some in-depth knowledge of the relationship between a team member's communication process and her outcome from the collaboration.

**Figure 2:**
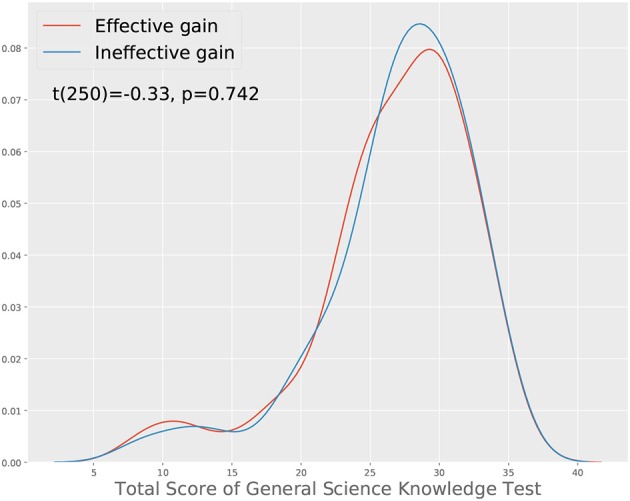
Comparison of the total scores from participants who gain effectively and ineffectively from the collaboration. A *t*-test shows that the two groups have similar contents-relevant science knowledge.

In the first approach, we started with the total score changes and examine how the CTPs are different in different groups. Specifically, we divide the participants into two groups, labeled effective gain and ineffective gain. Each participant in the effective gain group has a positive total score change while each in the ineffective gain group has a negative or zero total score change. One may notice that such a grouping may systematically penalize people with higher content-relevant knowledge, as they have a higher chance to have a correct initial response to a given item, so it is not possible to further improve. To ensure that we are considering people with comparable content-relevant knowledge, we removed the participants who correctly answered more than five of the seven questions in their initial response. After controlling on this, we have 151 and 101 participants in the effective gain and ineffective gain groups respectively. We verified that they have comparable content-relevant knowledge by comparing their performance in the general science knowledge test, as shown in Figure 2. The findings from this approach may be useful in informing the teaching or training of what features of the communication process lead to more effective collaboration outcomes.

In the second approach, we started with the communication process by clustering the participants based on their CTPs, then examined the total score changes in each of the clusters. To perform the cluster analysis, we flattened each CTP into a 16-dimensional vector by appending rows one after another, then calculated Euclidean distances based on the vectors between pairs of participants as a similarity measure of their communication processes. Based on this similarity measure, we first perform a hierarchical clustering analysis using Ward linkage (Ward, 1963) to cluster the participants and then examine the difference of the outcomes in terms of the total score change in different clusters. The findings from this approach can help to uncover similar patterns from the communication process that are associated with similar or different collaboration outcomes, which may also lead to meaningful feedback for a better teaching or training strategies for improving collaboration.

Both approaches may thus lead to actionable procedures in practice to diagnose issues in a computer-supported collaboration and provide feedback to better scaffold the collaboration. For example, after an online collaboration, if we found students who tend to respond to partners in a particular way often show poor collaboration outcomes, we can design coaching or training program to help them to change their ways of communication to ways that are more likely to lead to successful collaboration. The consistency of the findings from the two approaches will substantiate the efficacy of the CTP method for characterizing the communication process in a collaborative activity; whether these characterizations support effective feedback is beyond the scope of the present article.

## 4. Results

**Figure 3:**
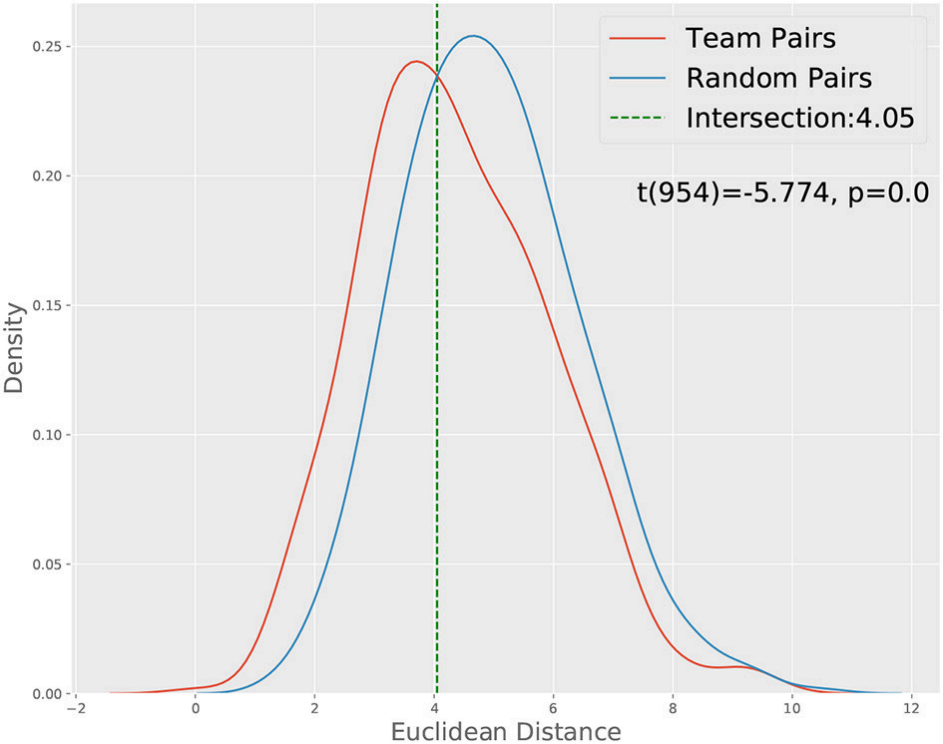
Distance distribution of team pairs and random pairs. A *t*-test show that the two distributions' means are significantly different.

Before we present the results corresponding to the two approaches described above, we would like first to check whether CTPs between team members are more similar compared to those between random pairs of participants. Given the interdependent nature of dyadic communication, we might expect the CTPs between the team members to be more correlated than those between random pairs of participants, which can serve as a check of the plausibility of the CTP approach. We carried out such an analysis based on the full dataset, i.e., without taking out those teams with more than three non-prompted conversations and show the results in Figure 3, where we compare the Euclidean distance between the CTPs from team members and random pairs. The result confirms our hypothesis of the interdependence of the communication between team members, which also lends support to the effectiveness of the CTP approach for characterizing the team member's communication process.

**Figure 4:**
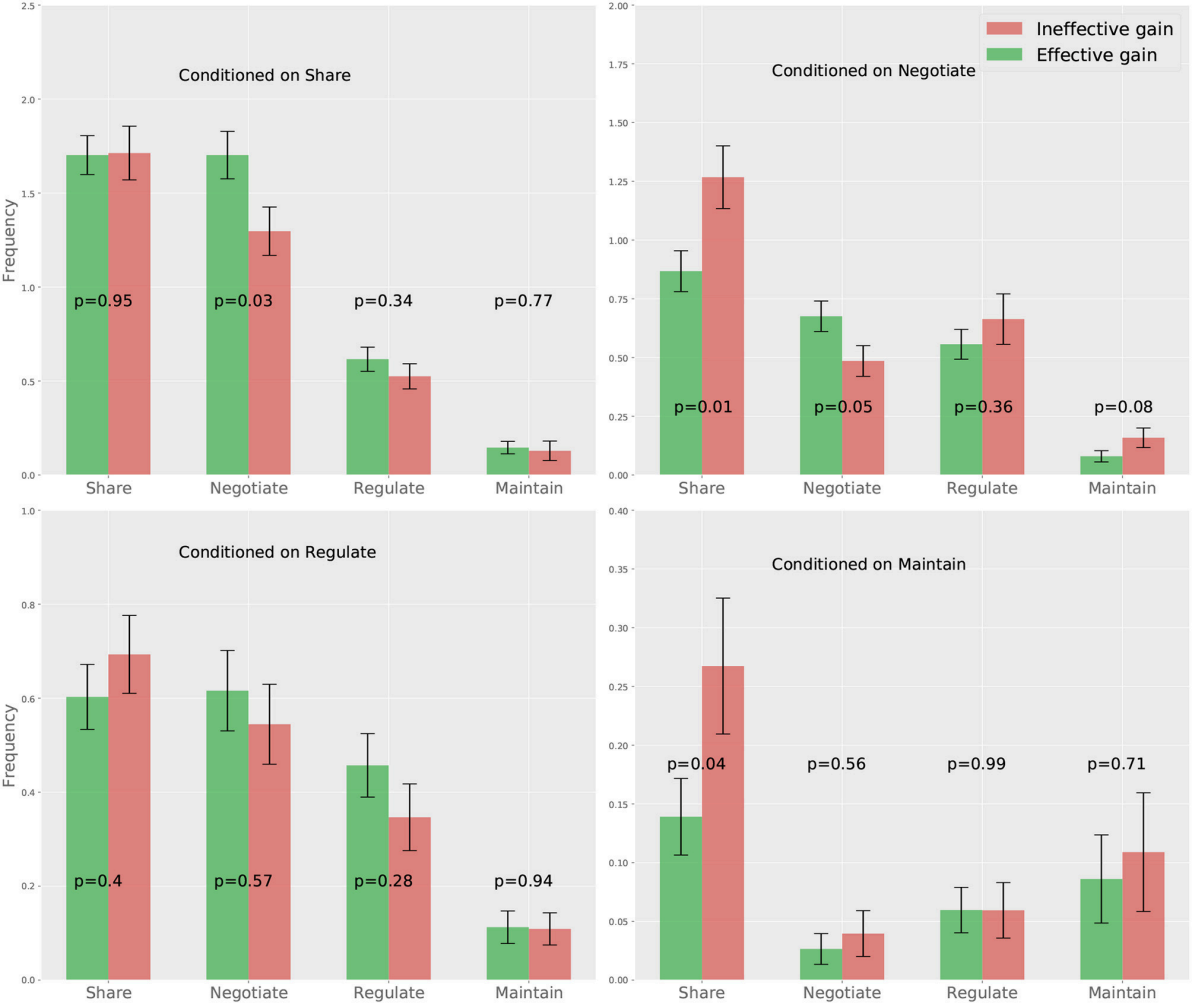
Mean and standard error of the CTPs correspond to the effective and ineffective gain groups. The p-values of pairwise *t*-tests for different CTP components are also presented.

The results from our first approach is shown in Figure 4, where we compare each element of the CTPs corresponding to the effective and ineffective gain groups via independent *t*-tests (2-tailed)^3^. The results show that the effective gain group has significantly more “negotiate” following the partner's “share” and “negotiate”, while the ineffective gain group shows significantly more “share” following the partner's “negotiate” and “maintain.” This findings suggests that a person is more likely to demonstrate improved performance if she shows more “negotiate” following her partner's “share” and “negotiate.” However, a person is less likely to get an improved response if she shows more “share” upon her partner's “negotiate” and “maintain.” This suggests the fact that negotiation is essential for gaining more from a collaboration, while excessively sharing information will contribute negatively, which is consistent with our earlier findings at the team level (Hao et al., 2016).

**Figure 5:**
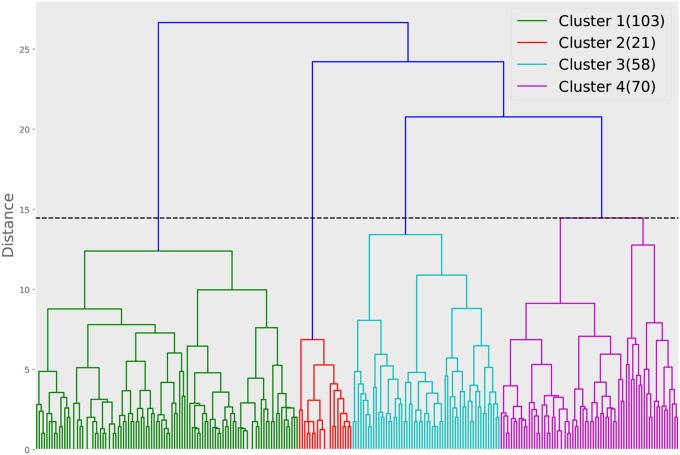
Dendrogram of the hierarchical clustering based on the Euclidean distance calculated from the CTPs. The horizontal dashed line is the distance cut corresponding to the elbow point of the inter-cluster distances. The numbers in the bracket in the legend show how many participants are in each of the clusters.

**Figure 6:**
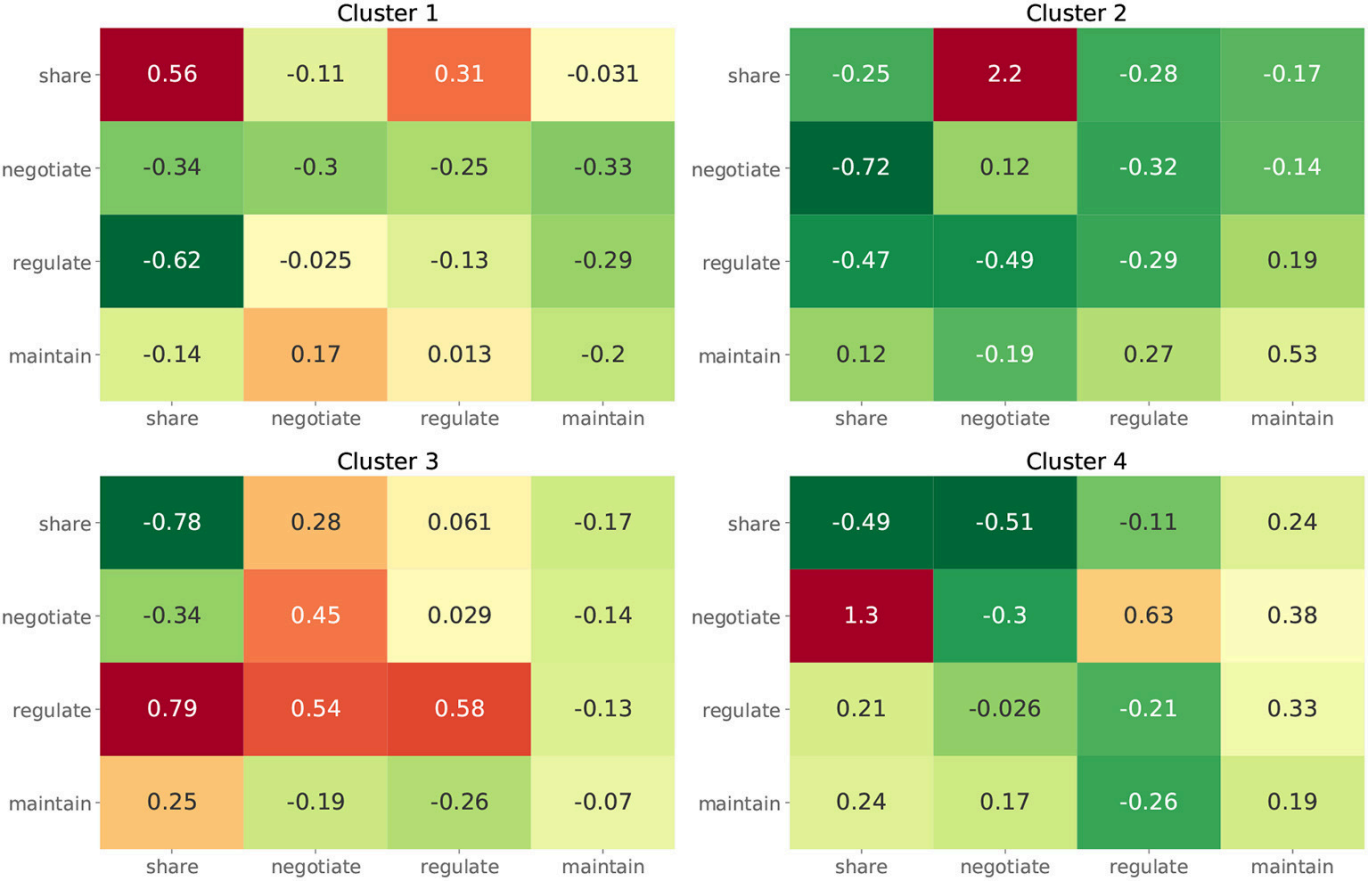
The effect size in terms of Cohen's d between the CPTs of participants from each cluster and from all participants.

**Figure 7:**
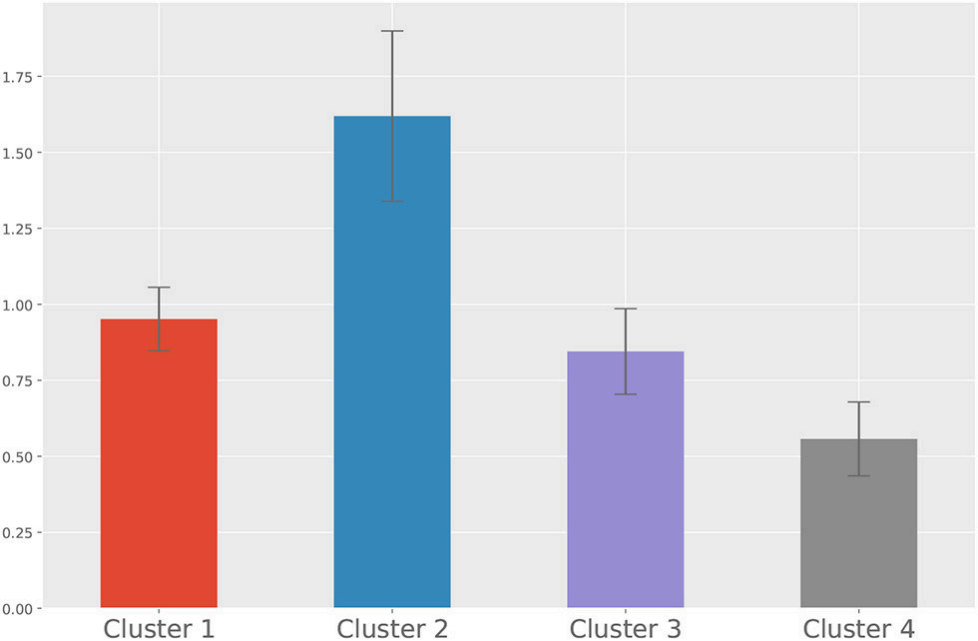
The means and standard errors of the total score changes from each cluster.

For the second approach, we show the dendrogram of the hierarchical clustering analysis in Figure 5. By examining the distance among the clusters at different levels, we noted that cutting the inter-cluster separations by the elbow point of the inter-cluster distances leads to four clusters. Each cluster is colored differently in Figure 5 and the number of members in each cluster is shown in the legend. To gain more insight into the differences among the four clusters, we compare their CTPs against the CTP of the overall participants by looking at the effect size in terms of Cohen's d. A positive value implies the people in that cluster show more conditional actions corresponding to that cell than the overall population, while a negative value implies the other way around. The results are shown in Figure 6. A general guideline (Sawilowsky, 2009) for interpreting the effect size is that a Cohen's d equal and greater than 0.8 is considered large effect. Then, in each panel of Figure 6, readers can identify how the corresponding cluster is different from the overall participants. Such a plot can give readers a general sense of the major difference between the clusters. Figure 7 further shows the total score changes in each cluster. The participants in cluster 2 show significantly more positive gain compared to people in other clusters. Connecting back to Figure 6, one can immediately identify the main feature of the cluster 3, e.g., participants show more “negotiate” actions when partners “share” information, which is consistent with the results from the first approach.

## 5. Conclusion and Future Work

In this paper, we introduced a CTP approach to characterize individual team member's communication process in computer-supported collaborations. Based on a large-scale empirical study and using two different approaches starting from the collaboration outcome and the communication process respectively, we show the CTP approach can effectively characterize aspects of one's communication process.

The purpose of the current study was to demonstrate the use of the CTP matrix rather than examine collaboration patterns in a controlled experiment. However, the results of applying CTP to the empirical study suggest that RM's one might try to negotiate while his/her team partner is sharing and negotiating ideas with him/her if he/she wants to gain more from the collaboration. Just sharing ideas seems less likely to help you gain more from collaboration, and even lead to worse outcomes if you do so while your partner is negotiating with you. This finding is consistent with our previous findings at the team level (Hao et al., 2016) and findings in the CSCL literature (Scardamalia and Bereiter, 1994; Stahl, 2006). Moreover, such findings can be incorporated into the teaching of collaborative problem solving skills, and can also be included into real-time feedback mechanisms for scaffolding collaboration.

Despite the effectiveness of CTP, the approach has several known limitations. The first is that it does not capture timing information that could contain useful information concerning, for example, the participation and engagement of the team members regarding their communication and collaboration. Timing is often strongly dependent on the specific task design, however, and its relationship with the other aspects of a collaboration can vary significantly from task to task. As such, a time-dependent version of the CTP with proper inclusion of timing data may provide a better characterization of the process in a given task situation but at the cost of reduced generalizability.

The second is that the CTP does not address possible random errors of the states, such as those introduced during the coding process. A future line of work that may help to improve along this direction may be the introduction of hidden states and emission probabilities to connect the hidden states to the observed states to accommodate the random errors, as Hidden Markov Models (Baum and Petrie, 1966).

The third is that the CTP may become very sparse if there are many coding categories and multiple participants. The average count of each element in the CTP scales down as 1/(*nk*^2^) with *n* as the number of team members and *k* as the number of coding categories. Users need to make sensible decisions regarding whether to use this method if the communication sequence is very short. A future line of work to address this limitation could consider latent variable modeling, such as factor analysis, though which one can identify a small set of factors to deal with the sparsity.

Finally, the communication process data used in this paper is relatively short, only about thirty turns on average when considering the first seven questions. Though some statistically significant effects have been detected at the subgroup level (thanks to a large number of participants), it does not allow us to reveal more details of each team member's process. In ongoing work, we have collected new data using a task hosted on the ETS Platform for Collaborative Assessment and Learning (Hao et al., 2017c). The new task elicits over 120 turns of communication in each team. We will report the findings based on the new data set in future work.

## Ethics Statement

The data collection is approved through ETS's IRB.

## Author Contributions

JH contributed to task development, data collection and analysis, research idea and method development, and presentation. RM contributed to research idea and method development, presentation and interpretation.

### Conflict of Interest Statement

The authors declare that the research was conducted in the absence of any commercial or financial relationships that could be construed as a potential conflict of interest.

## Footnotes

^1^In practice, the categories or states are assigned either by human coders or automated coding algorithms.

^2^Table 2 shows an empirical example of coded discourses.

^3^Note that multiple comparison happens in this case. As the Bonferroni correction is well-known to be too stringent for discovery-oriented studies, we adopted the False Discover Rate (FDR Benjamini and Hochberg, 1995) approach by setting the level of FDR to 0.2, which means we tolerate 20% of the discoveries to be false. At this FDR level, the adjusted p-value for significance is still 0.05 (which is a coincidence).
